# Facile Fabrication of Micro-Nano Structured Triboelectric Nanogenerator with High Electric Output

**DOI:** 10.1186/s11671-015-1001-5

**Published:** 2015-07-21

**Authors:** Feifei Zhang, Baozhang Li, Jianming Zheng, Chunye Xu

**Affiliations:** CAS Key Lab of Soft Matter Chemistry, Department of Polymer Science and Engineering, Hefei National Laboratory for Physical Sciences at the Microscale, University of Science and Technology of China, Hefei, 230026 People’s Republic of China

**Keywords:** Triboelectric nanogenerator, Electrospinning, Polyformaldehyde, Polytetrafluoroethylene, Electrochromic device, 84.60.-h, 81.40.-z, 73.61.Ph

## Abstract

In this article, a new method is used to fabricate a high-performance triboelectric nanogenerator (TENG), which is convenient and cost-effective. A polyformaldehyde (POM) film with novel structures is prepared through electrospinning and is combined with a polytetrafluoroethylene (PTFE) film to assemble micro-nano structured TENG. The short-circuit current (*I*_s_) and open-circuit voltage (*V*_o_) of the TENG are up to 0.4343 mA and 236.8 V, respectively, and no significant change is observed by applying different frequencies of external impact forces from 1 to 10 Hz. Finally, we successfully drive an electrochromic device (ECD) directly using TENG within just 2 min for the first time.

**Electronic supplementary material:**

The online version of this article (doi:10.1186/s11671-015-1001-5) contains supplementary material, which is available to authorized users.

## Background

Energy’s critical importance in social development and people’s lives is now universally recognized. A lot of technologies, such as photoelectric [1], pyroelectric [2], magnetoelectric [3], and piezoelectric [4], have been invented to collect energy in the environment, for example, in the form of light, heat, and motion. In recent years, triboelectricity is applied to a new type of generator named triboelectric nanogenerator (TENG) to harvest mechanical energy [5–9]. The TENG is efficient, flexible, and easy to fabricate, so it has aroused intense scholarly interest since its advent.

**Fig. 1:**
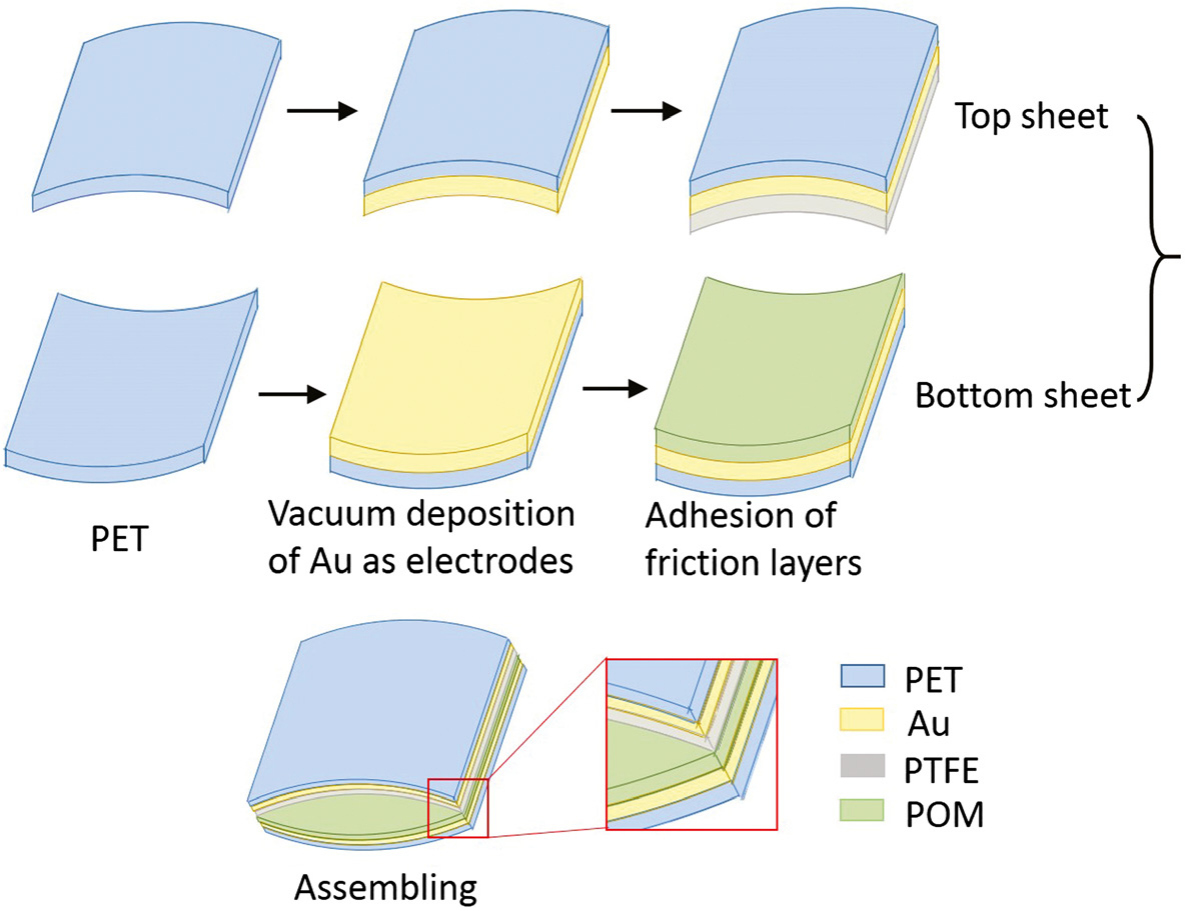
Schematic diagram of the TENG fabrication process

Typically, TENG is multilayered, consisting of friction layers and electrode layers (Fig. 1). It mainly utilizes the static charges generated during tribological process to induce electricity between the electrodes. Compared to the untreated friction surface, the micro and nano friction surface improved the output of TENG as it increased the area of friction layers and resulted in the generation of more electrostatic charges on the friction layers [10, 11]. Various methods have been tried to modify the friction surface such as ion beam etching [12], silicon template [10], anodic aluminum oxide template [13], and synthesis and assembly of nanoparticles and nanowires [11, 14]. However, these methods are either complicated or costly. Electrospinning is one of the novel fiber fabrication techniques because it is easy to produce continuous polymer fibers with diameters ranging from several nanometers to micrometers [15–17]. Using electrospinning to prepare nanowire-based TENG simplifies the preparation process and reduces the cost [18].

In this article, we propose a facile method to fabricate high-output TENG by preparing a micro-nano structured polyformaldehyde (POM) film through electrospinning as one friction layer and utilizing a polytetrafluoroethylene (PTFE) film as the other, which not only simplifies the fabrication process but also enhances the electric output of TENG. The open-circuit voltage (*V*_o_) of our prototype TENG reaches 236.8 V, and the short-circuit current (*I*_s_) is up to 0.4343 mA. Such high output current makes it sufficient to drive a homemade electrochromic device (ECD) directly. For these obvious advantages, TENG has potential application in the area of electronics, health care, and other practical applications.

## Methods

### Preparation of POM Film

To prepare the solution for electrospinning, 0.8 g POM (Yunnan Yuntianhua Co., Ltd., China) was dissolved into 9.2 g hexafluoroisopropanol (Aladdin Industrial Inc., China) in a 10-mL glass sample bottle [19]. The process was conducted by a NEU nanofiber electrospinning unit (Kato Tech Co., Ltd., Japan). One copper plate collector covered by aluminum foil was located 15 cm away from the needle tip of the syringe and was grounded. A high direct current voltage of 18 kV was applied between the needle tip and the copper plate collector, and the volumetric flow rate of the polymer solution was 0.8 mL/h. All the experiments were done at room temperature with a relative humidity of 55 %. The electrospinning process was finished after 4 h. The electrospun POM film was dried in a vacuum oven at room temperature overnight to remove the residual solvent.

### Fabrication of TENG

The typical fabrication process of TENG is depicted in Fig. 1. First, a thin layer of gold (100 nm) was deposited on two pieces of polyethylene terephthalate (PET) films (4 × 4 cm) (Dongguan Chang’an Chaoyuan Film Co., Ltd., China) by a sputter coater. Second, each PET film is adhered with a layer of double-sided adhesive tape on the gold side. Third, the PTFE film (4 × 4 cm) (Deqing Tonghe Plastics Research Institute, China) and electrospun POM film (4 × 4 cm) were respectively adhered onto the two arched PET films. Then, TENG was assembled by using adhesive tape to fix the two freshly prepared sheets along the straight sides with a width of 2 mm on each sheet.

### Characterization and Measurement

The morphologies of the electrospun POM film and untreated PTFE film were investigated using a field emission scanning electron microscope (FE-SEM) (JSM-6700F, JEOL, Japan). The electric output of TENG was measured using a digital multimeter (34410A, Agilent Technologies, Inc., USA). The external impact forces were provided by a vibration exciter (Baofei Vibration Instrument Plant, China).

## Results and Discussion

### Working Mechanism of TENG

**Fig. 2:**
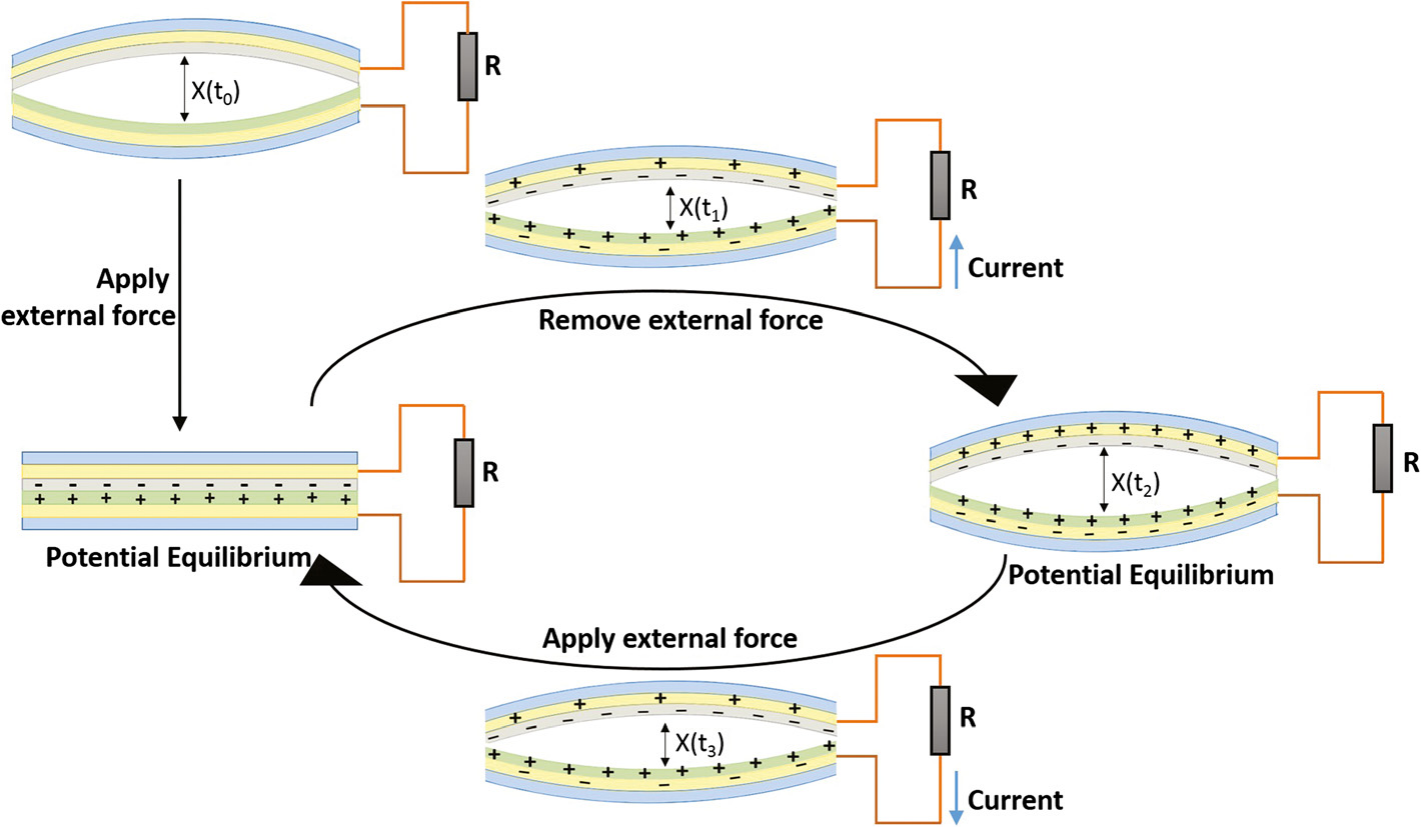
Working mechanism of TENG

The working mechanism of TENG is illustrated in Fig. 2. In the initial state, each layer of TENG is electrically quasi-neutral. After applying external compressive force for the first time, the arched TENG is deformed. The top sheet contacts the bottom one, and friction takes place between the contact surfaces because of surface roughness in microscale. As a result, the friction surfaces carry opposite electrostatic charges which will not bleed off or be neutralized immediately since both the polymer films and air are insulative. When removing the external force, the TENG tends to recover to arched state and the friction surfaces move apart. Meanwhile, the electric potential between the two electrodes varies with the relative displacement of the oppositely charged friction surfaces. Thus, there will be current in the load circuit until establishing potential equilibrium between the two electrodes. Applying the external force again breaks the former equilibrium and causes a reverse current to establish a new potential equilibrium. The mechanism is similar to a previously reported TENG [12]. Frequently applying and removing the external forces lead to more friction and cause the friction surfaces to come close and draw apart repeatedly, synchronized with the variance of electric potential difference between the two electrodes. Thus, there will be alternating pulsed current in the load circuit of the TENG.

Triboelectric properties of friction materials have a great influence on the performance of TENG. In our work, two low-cost polymers with quite different triboelectric properties were used as friction surfaces of TENG: PTFE is most likely to be negatively charged, and POM is easy to be positively charged by triboelectrification [20].

### Performance Analysis of TENG

**Fig. 3:**
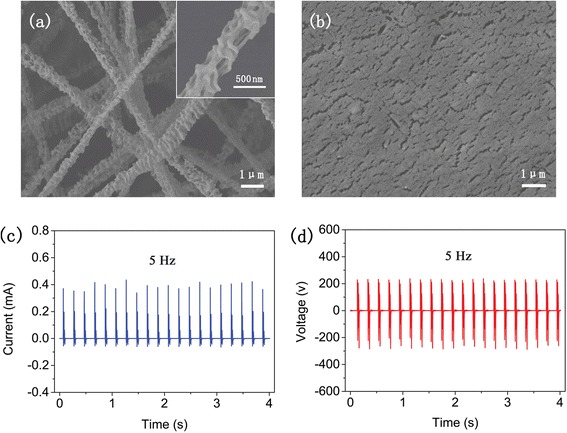
Micro-nano structured TENG with high electric output. **a** FE-SEM of electrospun POM; the *inset* shows the details of the fiber. **b** FE-SEM of PTFE. **c**, **d** *I*_s_ and *V*_o_ of the TENG under external impact forces of 5 Hz

In order to further increase the electric output of TENG, we prepared a micro-nano structured POM film since charges generated by friction are largely decided by the surface morphology of friction materials [21]. Utilizing electrospinning technology simplifies its preparation process, and the POM film with special structures is obtained through adjusting the electrospinning parameters. As shown in Fig. 3a, the POM fibers are randomly oriented with uniform diameters ranging from about 500 to 800 nm. Particularly, porous nanostructures are formed on the surface of the fibers. According to reported studies, rough surface with micro-nano structures and porous structures could enhance friction and increase contact area [10, 11, 22]. Thus, this novel micro-nano structure is supposed to enhance friction and to increase the electric output of TENG. In addition, the nanoscale cracks on the surface of the commercial PTFE film (Fig. 3b) are advantageous to the enhancement of friction. Consequently, more electrostatic charges will be generated and distributed on the surfaces of POM and PTFE after friction, which could help improve the performance of our TENG.

To investigate electric output of the micro-nano structured TENG, the *V*_o_ and *I*_s_ of the TENG under external impact forces of 400 N with a frequency of 5 Hz were measured, as shown in Fig. 3. It shows that the peak *I*_s_ is 0.4343 mA and the peak *V*_o_ is 236.8 V. The current density of 27.14 μA/cm^2^ is more than eight times higher than that of TENGs fabricated with PTFE and chemically modified titanium dioxide or surface-modified aluminum [14, 23], which demonstrates that our facile prepared TENG has high current output.

**Fig. 4:**
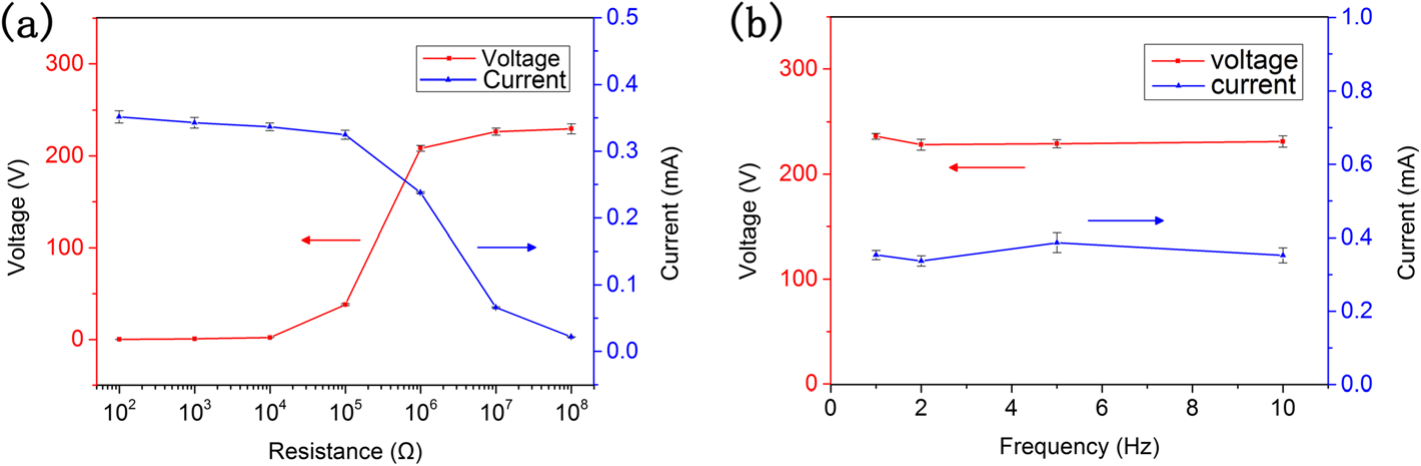
Electric output performance characterization of TENG. **a** Circuit current and voltage under variable load resistances. **b** *I*_s_ and *V*_o_ under different frequencies

To further investigate the performance of TENG, we measured the electric output of TENG when it was connected to variable load resistances, ranging from 100 Ω to 100 MΩ, as shown in Fig. 4a. It is found that the current of the circuit decreases with the increasing of load resistance while the voltage has a reverse trend. The instantaneous power on the load will reach a maximum value of 57.18 mW at a load resistance of ~1 MΩ (see Additional file 1).

To study the influence of external force frequency on the electric output of TENG, further experiments under external impact forces of 400 N with frequencies of 1, 2, and 10 Hz were conducted (see Additional files 2 and 3). The output *I*_s_ and *V*_o_ and their standard deviation are plotted against frequency in Fig. 4b. It shows that the peak values under different frequencies of external impact forces are approximately the same, indicating that *I*_s_ and *V*_o_ are not affected by frequency. This phenomenon seems different with some articles [10, 24] reporting that the output of TENG varied with frequency. It is reasonable, however. According to a theoretical study [25],

1 $$ {I}_{\mathrm{s}}=\frac{S\sigma {d}_0v}{{\left({d}_0+x\right)}^2} $$

2 $$ {V}_{\mathrm{o}}=\frac{\sigma x}{\varepsilon_0} $$

where *S* and *σ* are the area and charge density of the friction surface, respectively, *x* is the distance of the top and bottom sheets, *v* is the rate of relative displacement of top and bottom sheets, *d*_0_ is a constant related to thickness and relative dielectric constant of friction films, and *ε*_0_ is the vacuum dielectric constant. It can be learnt that *I*_s_ and *V*_o_ have no direct correlation with frequency, but on the condition that frequency changes *x* or *v*, it may affect *I*_s_ and *V*_o_ indirectly. In this work, the experimental phenomenon is attributed to the way in which external forces were applied. Pulse signal was used to modulate the vibration exciter so that frequency did not cause much change to *x* and *v*. The detailed and precise influence still needs further research.

**Fig. 5:**
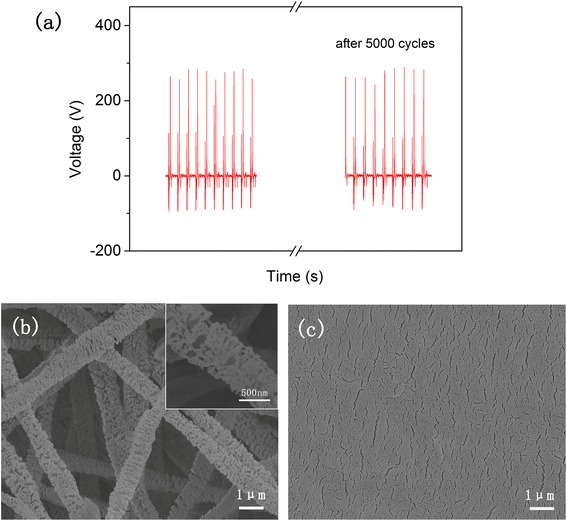
Electric output stability of TENG. **a** *V*_o_ of the TENG before and after 5000 cycles. **b** FE-SEM of POM after 5000 cycles. **c** FE-SEM of PTFE after 5000 cycles

Moreover, *V*_o_ of the TENG was measured after more than 5000 cycles to test the electric output stability (Fig. 5). It shows that there is no obvious decline (Fig. 5a). This might be due to the retained morphologies of POM and PTFE that have enhanced friction (Fig. 5b, c).

**Fig. 6:**
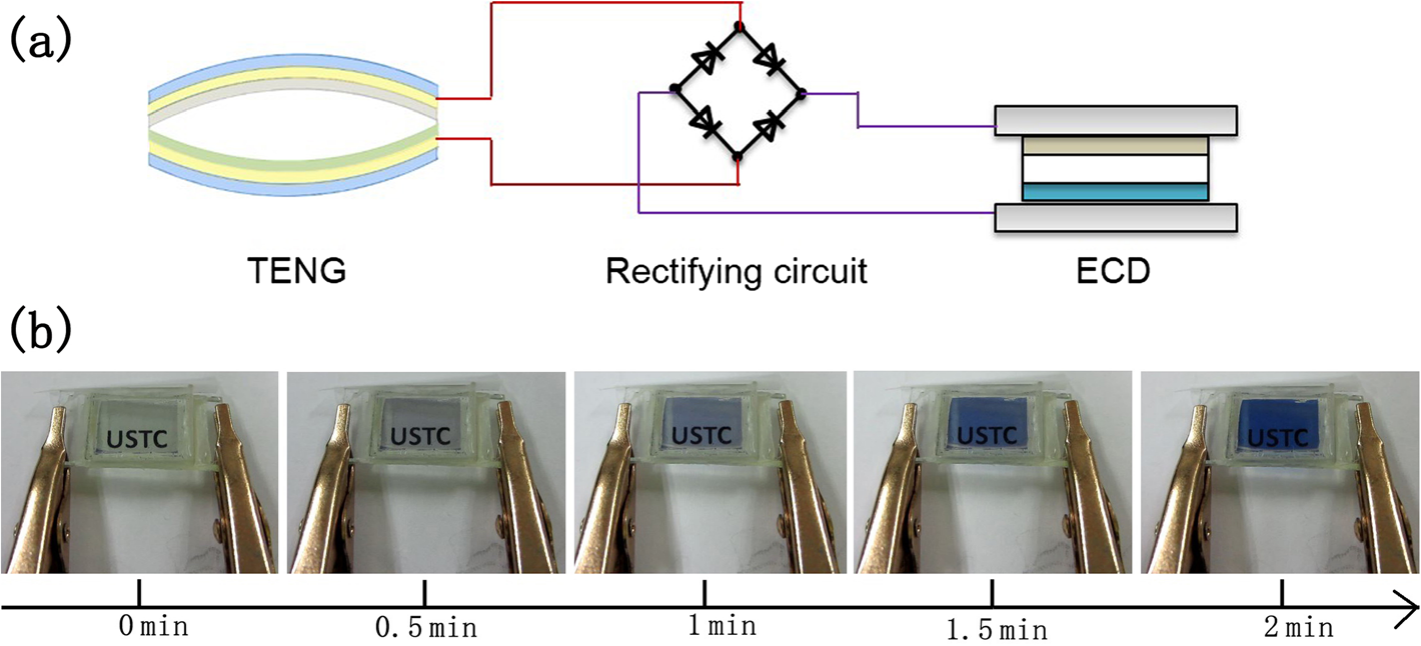
TENG as a power source to drive ECD. **a** Schematic diagram of the circuit. **b** Color change of the ECD driven by TENG

The high and stable electric output of the TENG implies its application to low-energy electronics, for example, ECD, which changes light transmission properties in response to voltage. We connected the TENG to a homemade ECD (1 × 1 cm) [26] through a rectifying circuit directly. By applying excitation impact forces of 10 Hz on the TENG continuously, the ECD switched from bleached state to colored state in 2 min (Fig. 6). This implies the promising potential of TENG in charging batteries and powering light-emitting diodes, liquid crystal displays, and some portable electronics.

## Conclusions

In summary, we report a new method to fabricate high-output TENG with an easy and cost-effective approach, which contributes to the practical application of TENG. Our experimental results show that the electric output of the fabricated TENG is rather high as *I*_s_ is up to 0.4343 mA and *V*_o_ reaches 236.8 V, and the output current can be directly used to drive homemade ECD. The good performance and facile fabrication of TENG indicate its potential application in the area of electronics, health care, and other practical applications, especially portable electronics. By further optimizing structure, we believe that it will afford a broader range of applications.

## Additional files

Additional file 1:
**Output power dependence on the resistance of external load.**
Additional file 2:
**External impact forces with different Frequencies.**
Additional file 3:
***I***_***s***_ **and** ***V***_***o***_ **of the TENG under different frequencies.**

## Abbreviations

ECD: electrochromic device
FE-SEM: field emission scanning electron microscope
POM: polyformaldehyde
PTFE: polytetrafluoroethylene
TENG: triboelectric nanogenerator
