# Supplementary figures and images for: Facile Fabrication of Micro-Nano Structured Triboelectric Nanogenerator with High Electric Output
Source: Nanoscale Res Lett. 2015 Jul 21;10:298. doi: 10.1186/s11671-015-1001-5 (PMC4508282; doi:10.1186/s11671-015-1001-5)

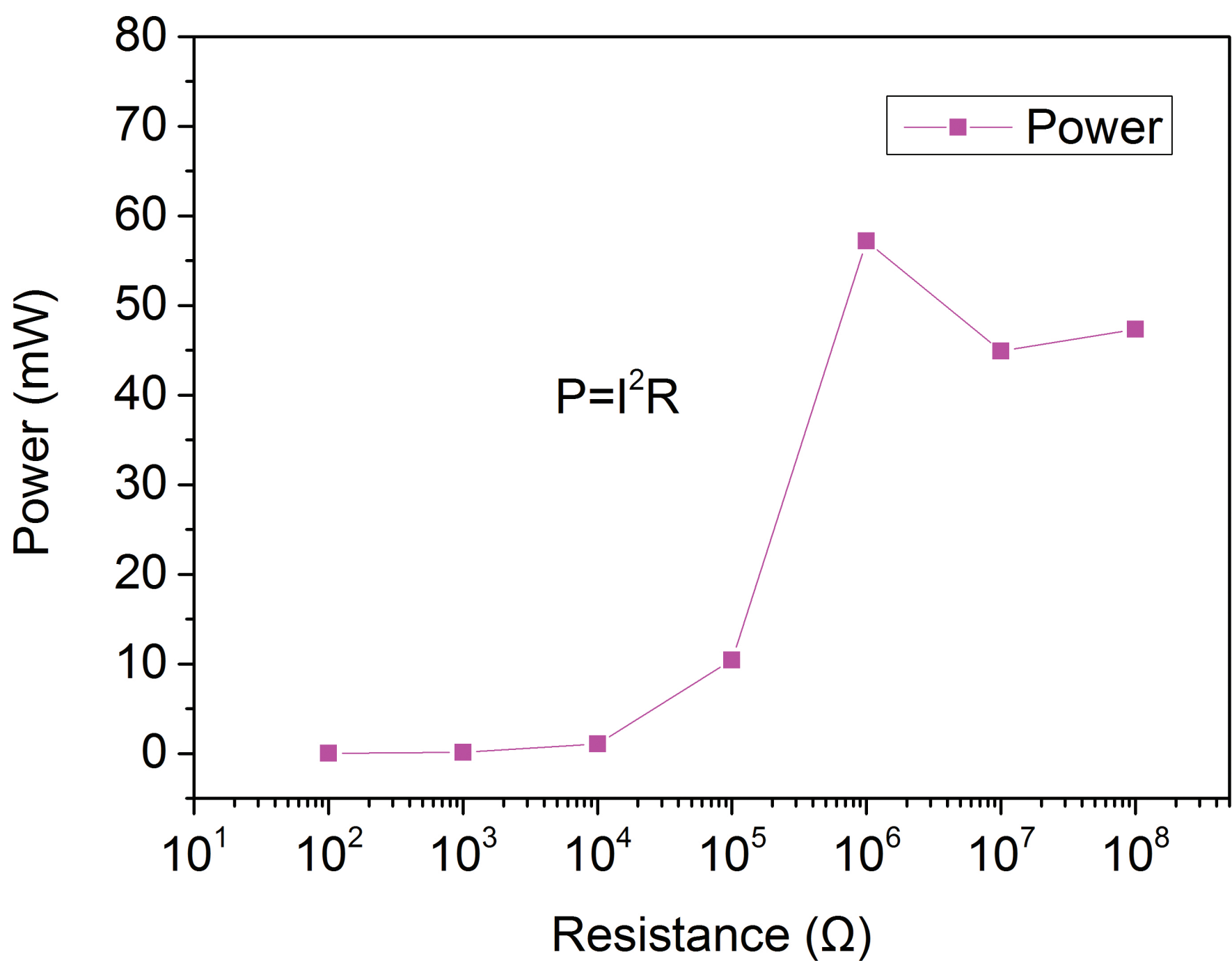

Output power dependence on the resistance of external load.

Supplement: Additional file 1: — Output power dependence on the resistance of external load. [file 11671_2015_1001_MOESM1_ESM.pdf]

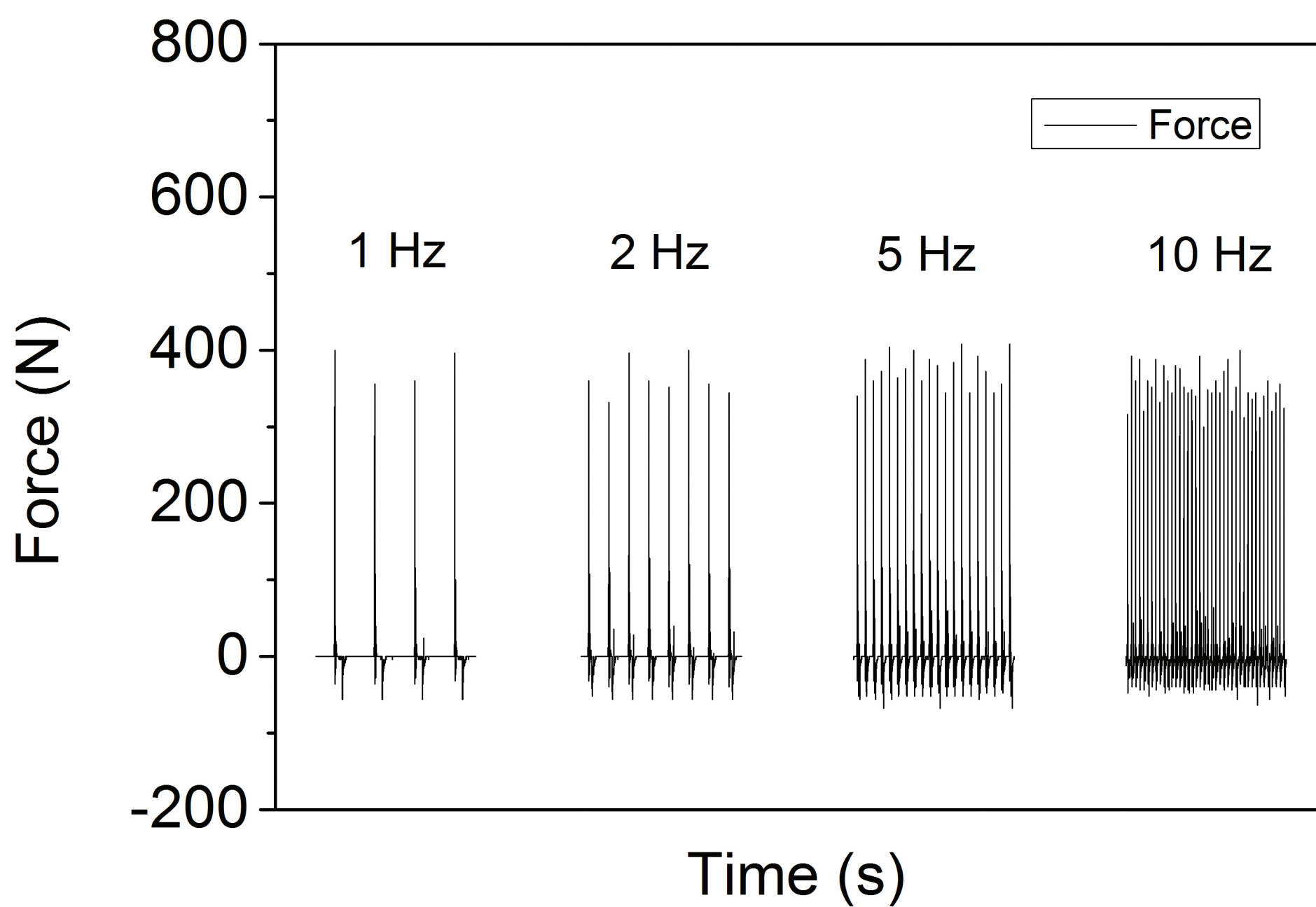

External impact forces with different Frequencies.

Supplement: Additional file 2: — External impact forces with different Frequencies. [file 11671_2015_1001_MOESM2_ESM.pdf]

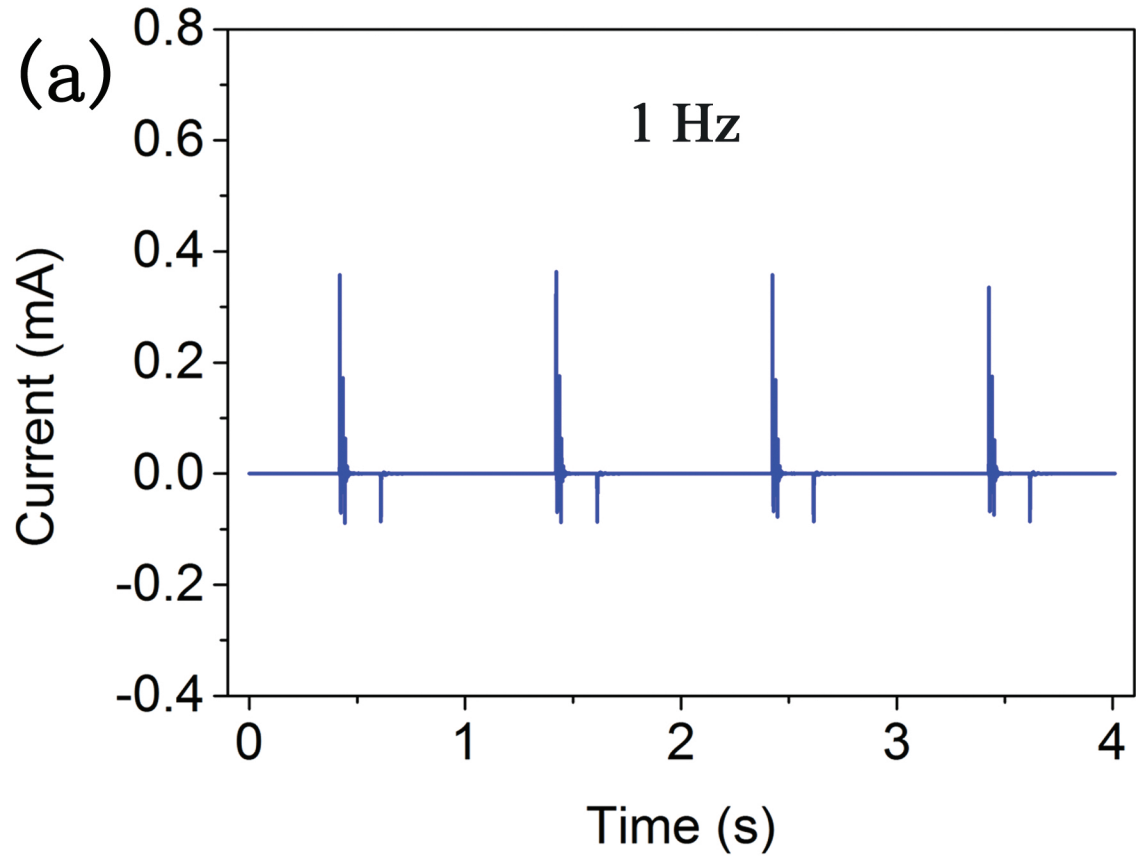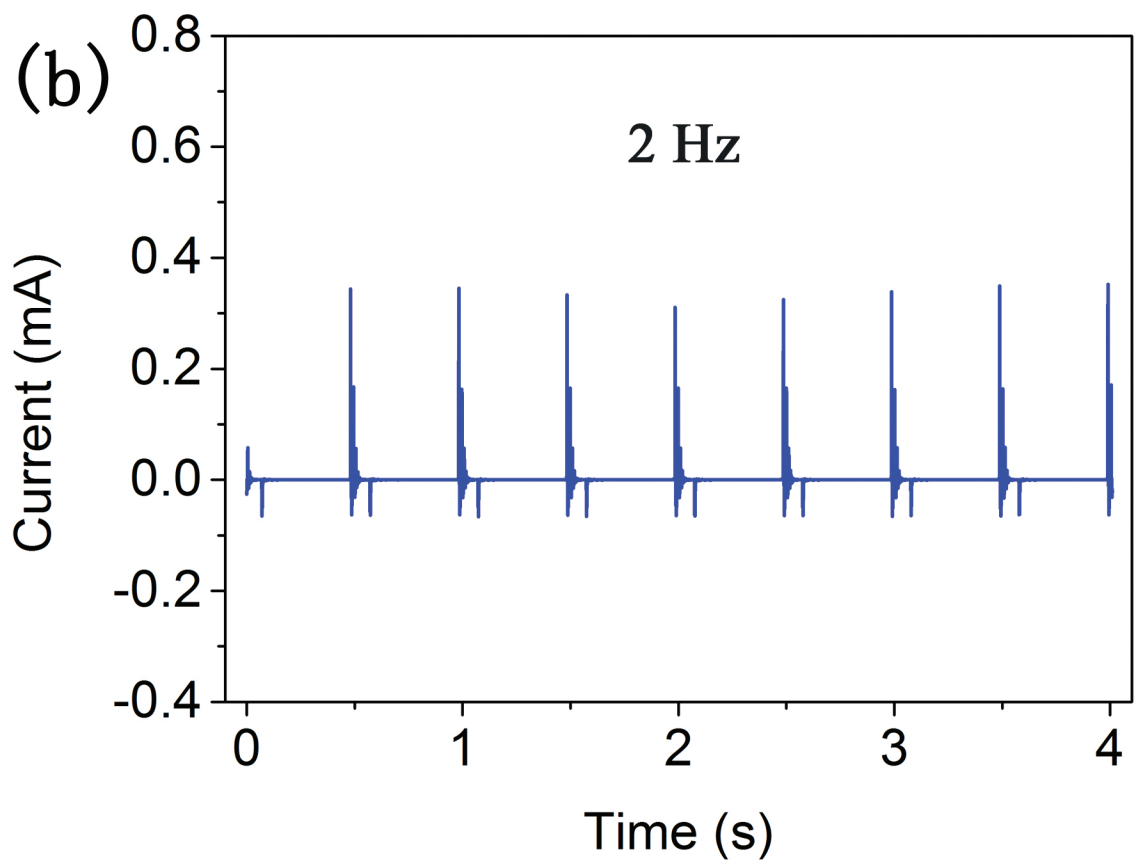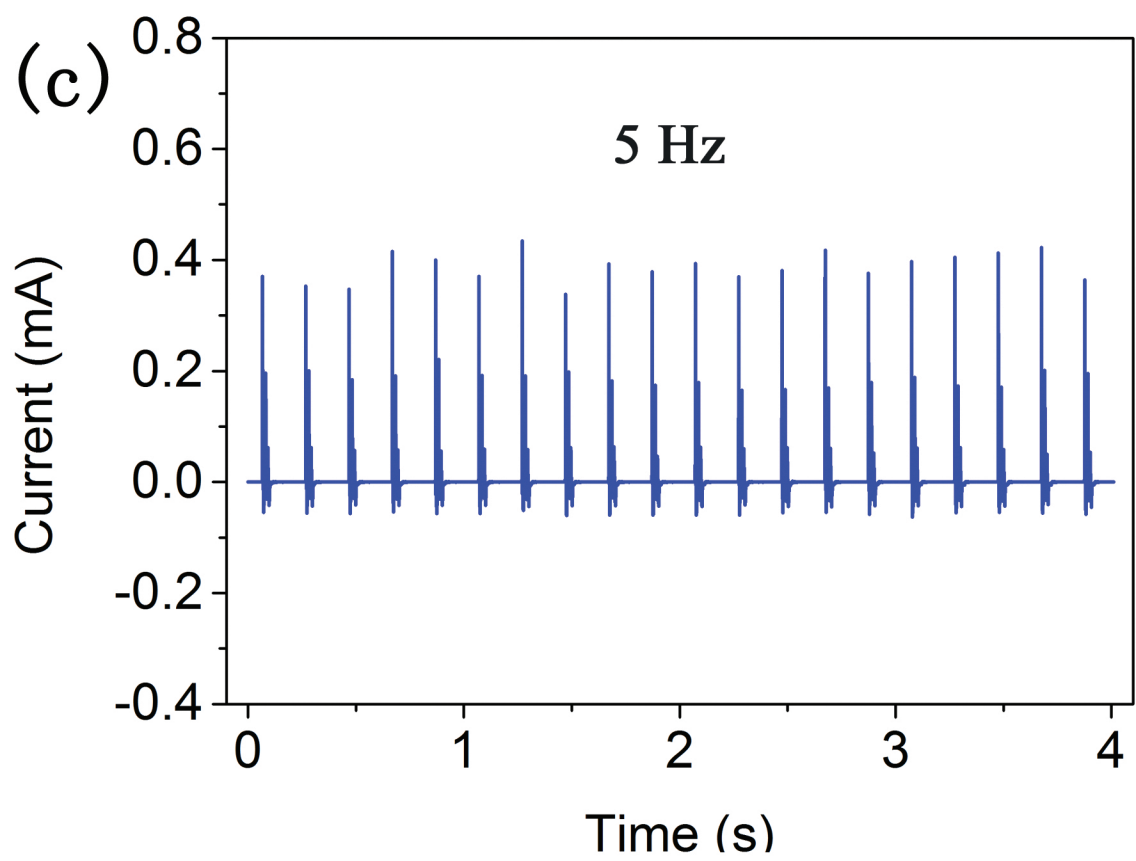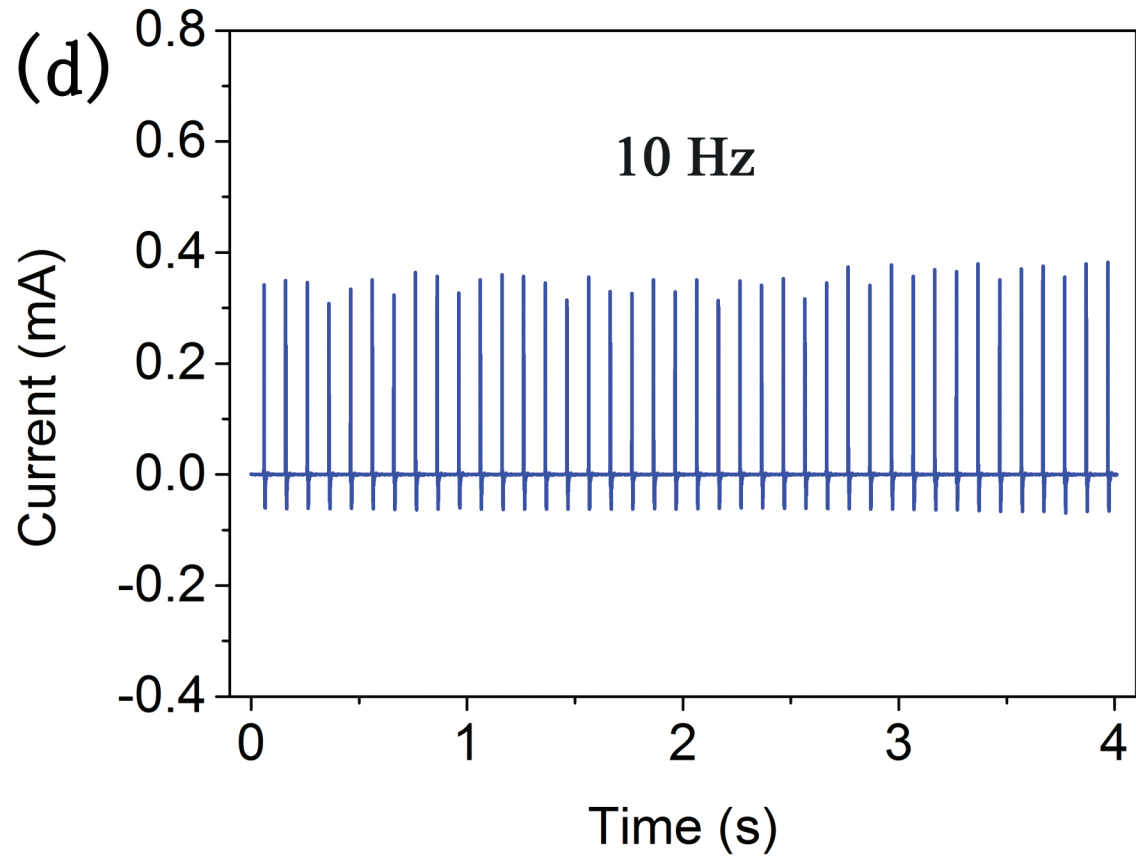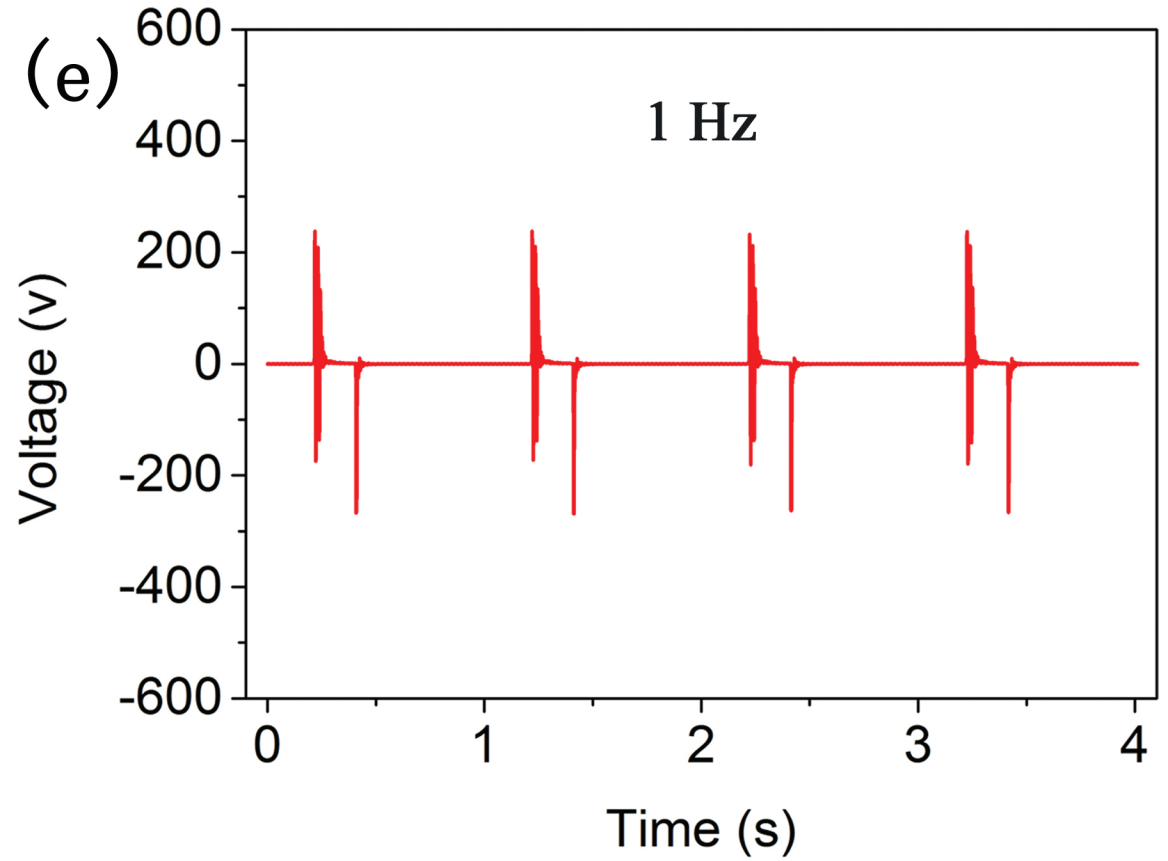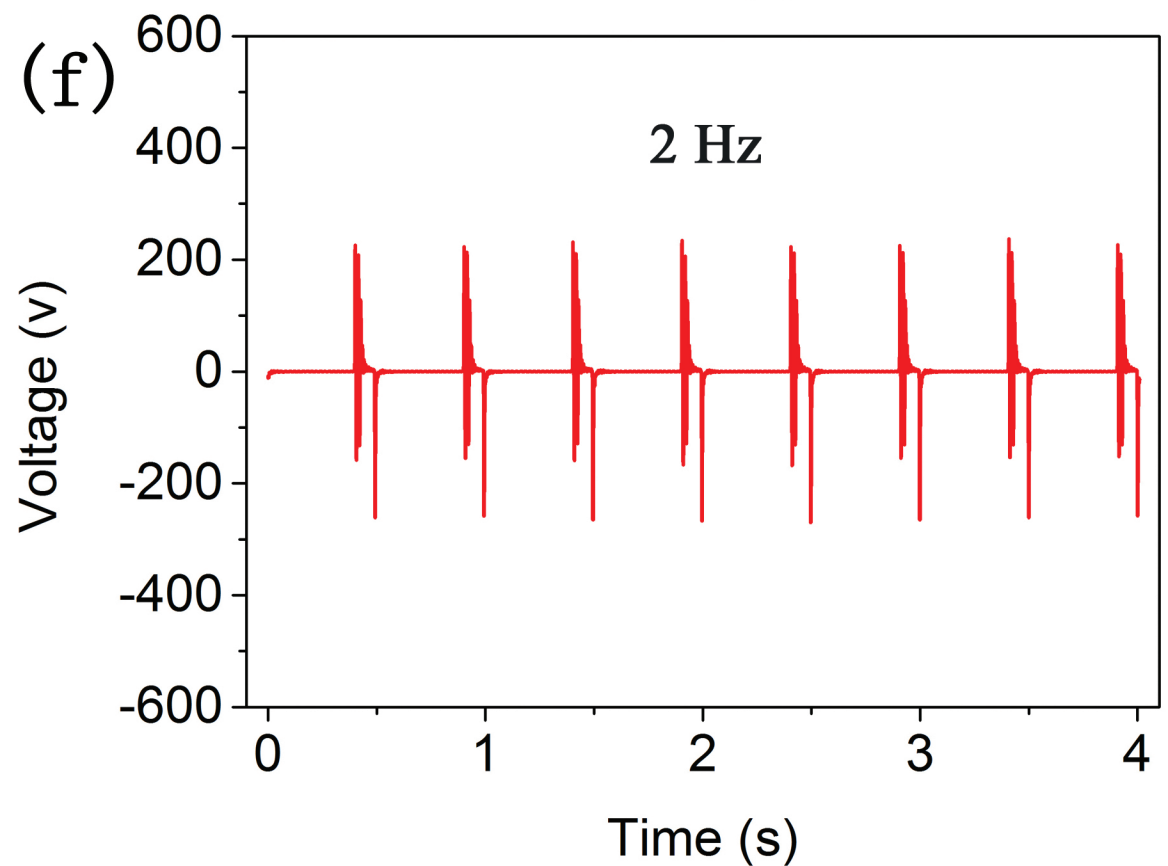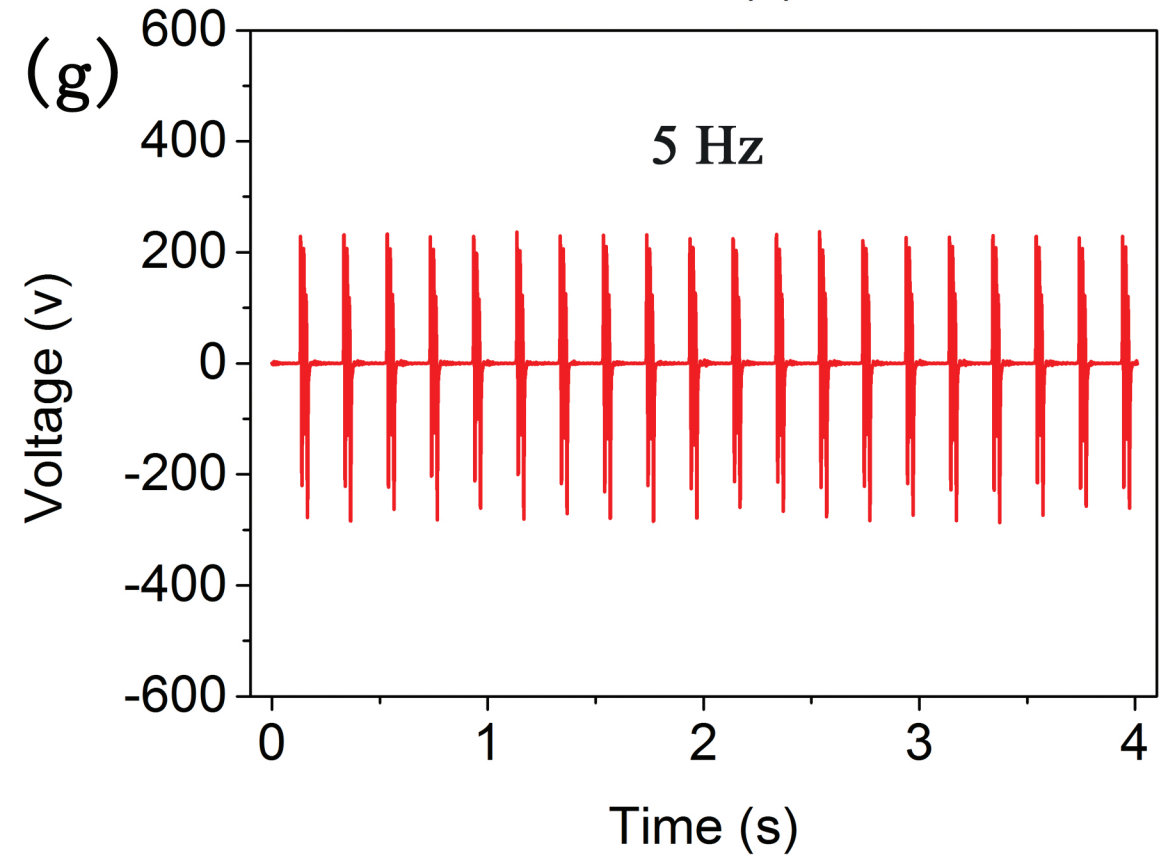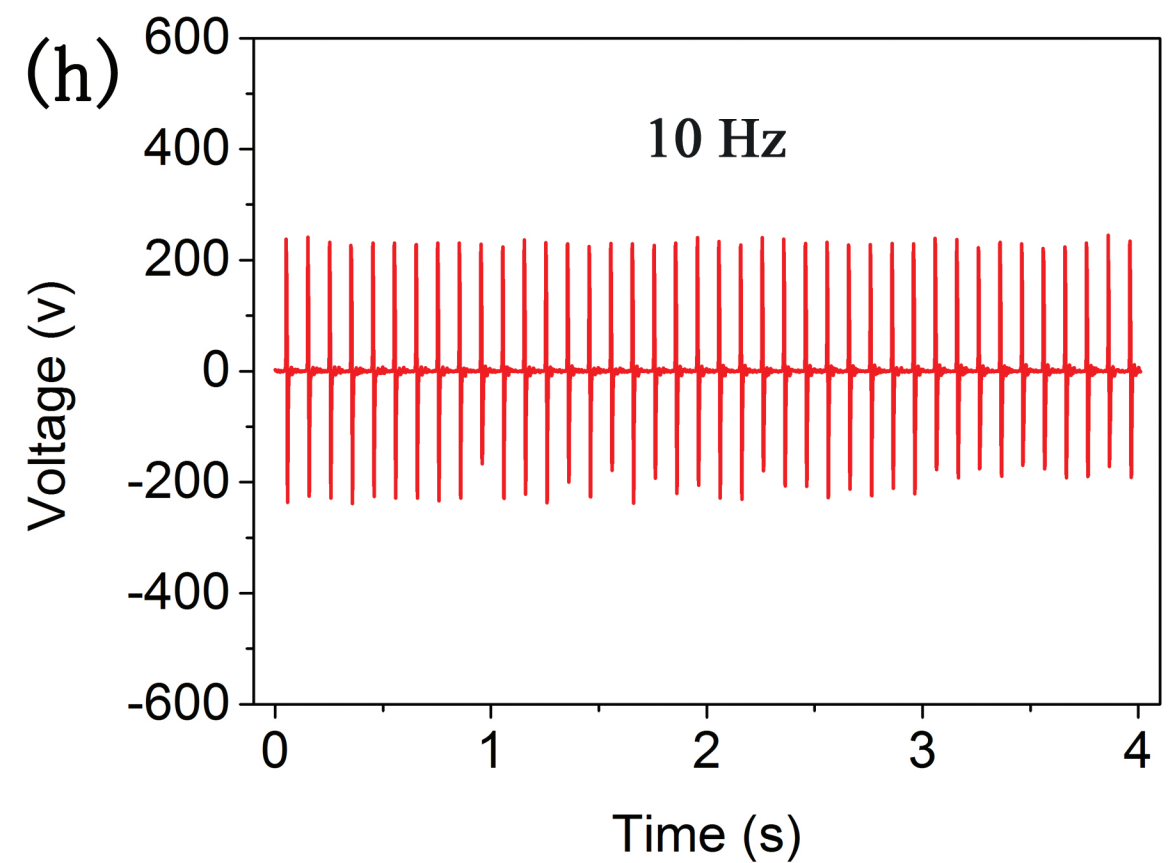

$I_s$  and  $V_o$  of the TENG under different frequencies.

Supplement: Additional file 3: — I s and V o of the TENG under different frequencies. [file 11671_2015_1001_MOESM3_ESM.pdf]
